# HeSARIC: A Heterogeneous Cyber–Physical Robotic Swarm Framework for Structural Health Monitoring with Augmented Reality Representation

**DOI:** 10.3390/mi16040460

**Published:** 2025-04-13

**Authors:** Alireza Fath, Christoph Sauter, Yi Liu, Brandon Gamble, Dylan Burns, Evan Trombley, Sai Krishna Reddy Sathi, Tian Xia, Dryver Huston

**Affiliations:** 1 Department of Mechanical Engineering, University of Vermont, Burlington, VT 05405, USA; alireza.fath@uvm.edu (A.F.); dylan.burns@uvm.edu (D.B.);; 2 Department of Mechanical Engineering, Indian Institute of Technology Madras, Chennai 600036, India; 3 Department of Electrical and Biomedical Engineering, University of Vermont, Burlington, VT 05405, USA; txia@uvm.edu

**Keywords:** heterogeneous swarm, cyber–physical system, structural health monitoring, semantic crack segmentation, culvert inspection, confined space inspection

## Abstract

This study proposes a cyber–physical framework for the integration of a heterogeneous swarm of robots, sensors, microrobots, and AR for structural health monitoring and confined space inspection based on the application’s unique challenges. The structural issues investigated are cracks in the walls, deformation of the structures, and damage to the culverts and devices commonly used in buildings. The PC and augmented reality interfaces are incorporated for human–robot collaboration to provide the necessary information to the human user while teleoperating the robots. The proposed interfaces use edge computing and machine learning to enhance operator interactions and to improve damage detection in confined spaces and challenging environments. The proposed swarm inspection framework is called HeSARIC.

## 1. Introduction

This research integrates several technologies such as robots, sensors, and a swarm of microrobots with augmented reality (AR) headsets using machine learning and computer vision in particular applications of damage analysis and monitoring. The framework will provide infrastructure inspectors with a tool for further routine investigation of difficult-to-reach areas, which is critical in structural health monitoring (SHM). 

Structural health monitoring enables continuous monitoring without damage to the structures and assists in reducing maintenance costs and the dependency on regular inspections [1]. The activities of SHM are classified into five levels—1. detection; 2. localization; 3. assessment; 4. prognosis; and 5. remediation [2]—and the effective strategies to employ the SHM are based on the inspection scale, response type, behavior, computation, feedback, excitation, and domain [3].

Here, the first steps in proposing a structural cyber–physical system (SCPS) are presented for the integration of SHM data to notify control systems. In the case of a disaster, this could reduce the chance of a structural failure [4]. One of the main challenges in designing the CPS for SHM-based applications is that specific requirements are necessary for each application. Some of these common requirements are reliable low-power communication, reliable data communication, remote access, heterogeneous WSN, data collection specifications, requirements on the sensors, and sampling [5].

Additionally, applying the concepts of swarm intelligence (SI) to a CPS can improve its robustness, scalability, and adaptability. Nonetheless, the challenges of incorporating such systems in real-world applications are the issues of reliability, real-time processing, and data transfer [6]. Cyber–physical approaches increase the supervisory efficiency of the IoT sensor monitoring of the environment, which presents remote access to the parameters [7]. As an example of the CPS, Sanneman et al. [8] developed a multi-crawling and swimming robot with origami flowers with LEDs and pouch motors called the Robot Garden. This platform shows rapid fabrication techniques and operates either with a graphical user interface or autonomously for educational purposes.

Moreover, the Human-In-The-Loop (HITL) robotic deployment for hazardous inspections can be conducted through efficient CPS cooperation using a heterogeneous Symbiotic Multi-Robot Fleet (SMuRF) for symbiotic autonomy and autonomy as a service [9].

At present, the use of augmented reality devices as user-centric interfaces is growing in applications of structural health monitoring [10], maintenance [11], robotics [12], education [13], automotives [14], and healthcare [15]. Extending human perception through AR and the use of sensors deployed on mobile robots enhances the way engineers inspect infrastructure. Such interfaces for human–machine interactions assist in making informed decisions for SHM [16]. For this purpose, a variety of microrobots controlled by AR were developed and investigated for use in confined spaces [12]. Incorporation of these novel microrobotic systems into SHM applications is essential for advancing intuitive confined-space exploration and inspection.

For smart-home applications, CPS-model-based approaches are utilized for controlling the devices via voice [17], and the Cyber–Physical Human Centric System (CPHCS) has been suggested for optimizing energy consumption while considering individual physiology to enhance thermal comfort [18].

In addition, as the need for human–robot collaboration grows, considering humans as an integral part of the CPS, called the Collaborative Robotic Cyber–Physical System (CRCPS), improves efficiency and safety. The Anthropocentric Cyber–Physical System (ACPS) approach can be used where human input is necessary for training the robots [19].

In this paper, a framework that includes a swarm of microrobots and robots with machine learning and edge processing, called the Heterogeneous Swarm Augmented Reality Robotic Inspection Cyber–Physical System (HeSARIC), is proposed for structural health monitoring. The applications of this framework in SHM applications are investigated through function tests of swarm semantic segmentation of cracks, swarm monitoring of structural deformations, swarm culvert inspection, Quadruped Robot Dog (QRD) wall inspection, and pump evaluation.

## 2. Materials and Methods

Despite the advancements in robotics, microrobotics, sensing technologies, and wireless networks, the challenges of applying low-cost application-based SHM inspections prevent many industries from incorporating them into their applications.

**Figure 1:**
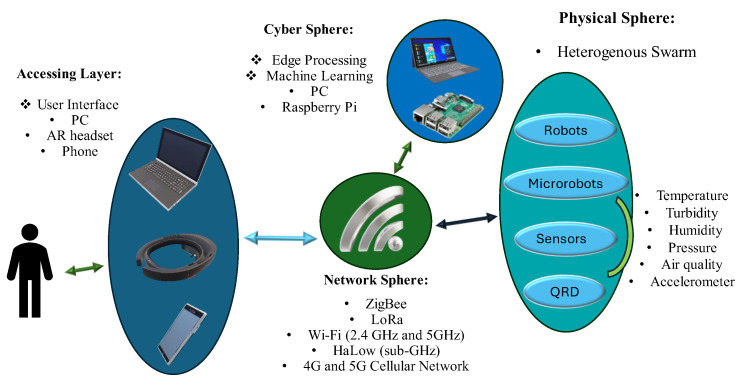
Schematic of CPS components with suggested applicable hardware.

CPS approaches are required to solve challenges in specific applications. In this section, a summary of the technologies required for each application, including the network connectivity options, is presented in Figure 1.

Contrary to specific hardware advancements, in CPS approaches, the efficient functioning of all the components as a whole is of importance.

As the perception layer, one important consideration in user-centric CPS approaches is that networks may contain numerous wireless sensors and robotic systems that transmit the information back to the user. As a result, the operator might become overloaded with raw information. Considering this fact, the Gate Control Theory of pain, proposed by Melzack and Wall [20], could be applied in a similar analogy to the CPS framework to control the information. In this method, the perception of pain is modulated by a gate. Likewise, in the CPS, the information the user receives through the network can be modulated by designing special user interfaces in AR [21]. Designing the appropriate interface for displaying the information and controlling the system will enhance the operator’s interaction with the hardware.

The network component of CPS is essential in those SHM applications in which the connectivity is challenging, such as culverts that are electromagnetically lossy. Therefore, the choice of network connectivity specifications and their limitations need to be investigated. In these conditions, the efficiency of the data transmission is a factor that determines the protocol and the data size required for the SHM.

Moreover, accessibility to the inspection site affects the hardware as damage to the structure may exist in areas that humans cannot inspect. Hence, utilizing robots of a variety of sizes and locomotion mechanisms can assist in reaching the areas of interest while sensing and processing the necessary parameters required by the SHM.

**Figure 2:**
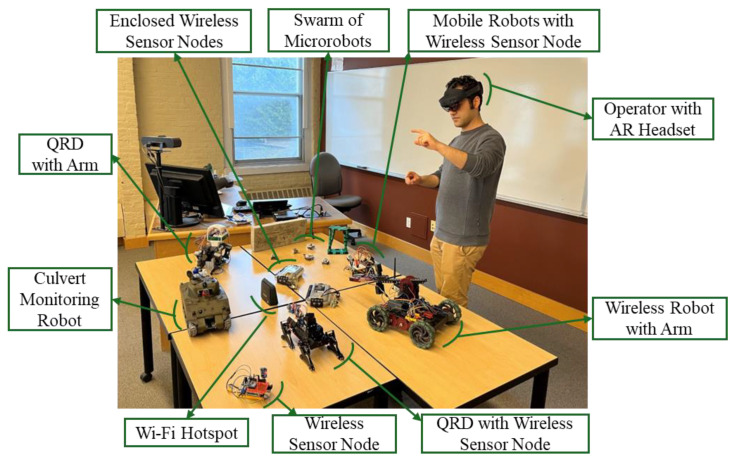
Heterogeneous swarm of a distributed wireless robotic and sensing augmented reality inspection cyber–physical system.

Figure 2 demonstrates some of the technologies incorporated in this study.

In the following sections, several robotic systems and devices are proposed for the inspection of challenging environments by providing methods to enhance accessibility, damage detection, efficient data transmission, and operator interaction with the CPS.

### 2.1. Swarm Microrobot Steering and Structural Inspection in AR

**Figure 3:**
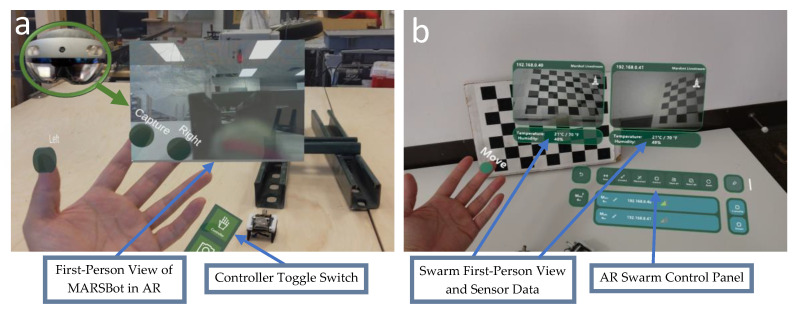
(**a**) AR interface to control the microrobots. (**b**) Early development of an AR interface for swarm microrobot detection in a network and for displaying their sensor information.

A major hurdle in SHM is the necessity for the inspection of confined spaces. While the use of robots for inspection has been widely explored, the challenges of fabricating microrobots for untethered inspection has hindered their advancement. To aid in this, multiple low-cost microrobots called MARSBots [12] were developed using an ESP32-Cam (Ai-Thinker, Shenzhen, China) with an eccentric mass mounted on an LCD 3D-printed flexible legs and a rigid board holder to connect to the board. The microrobot moves based on the bristle bot locomotion mechanism and steers to the left and right based on the bending of the fingers while the user is wearing an AR headset or sending commands using a PC. The AR headset (HoloLens 2, Microsoft, Redmond, WA, USA) detects all the microrobots in the swarm connected to the network, displays their sensor data information, and steers them in AR (Figure 3).

The logic is that once a connection is established with a detected MARSBot, the system immediately creates a class instance representing that MARSBot and generates a toggle switch indicating its status, e.g., signal strength, activated state, toggled state, etc. This class contains methods for sending commands to the specific MARSBot. When a movement command is issued, the system checks which toggle switches are currently active and sends the command only to those MARSBots whose switches are toggled on.

The proposed system allows for the inspection of hard-to-reach areas, structural changes, the integration of temperature and humidity sensors, and the incorporation of artificial intelligence for the semantic segmentation of cracks in damaged structures.

#### 2.1.1. Swarm Microrobotic Steering and Structural Inspection

**Figure 4:**
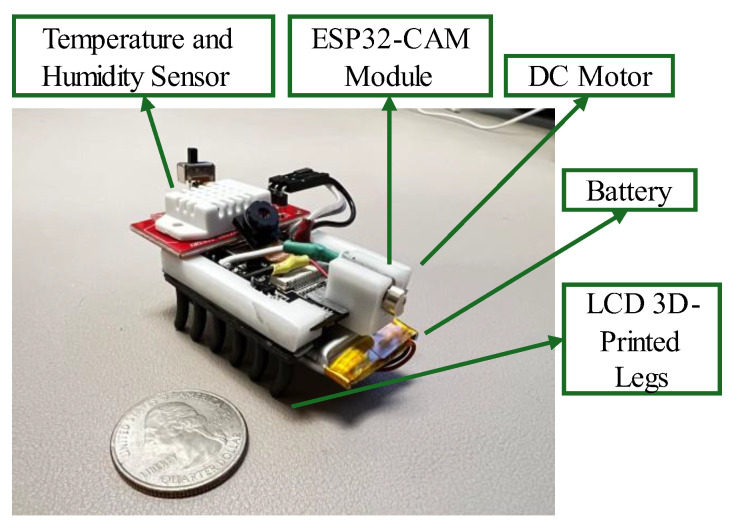
A MARSBot with a temperature and humidity sensor.

To receive further sensing data and to improve battery life, the MARSBot [12] was upgraded with a higher capacity battery and a humidity and temperature sensor as depicted in Figure 4.

**Figure 5:**
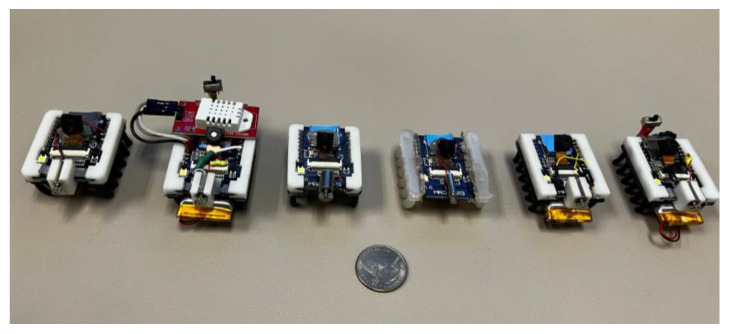
Swarm of microrobots with diverse characteristics.

A swarm of microrobots with different specifications and characteristics has been developed that can be employed based on the application’s specifications (Figure 5). The proposed microrobotic swarm system can be integrated into the HeSARIC framework in addition to other application-based robotic systems.

**Figure 6:**
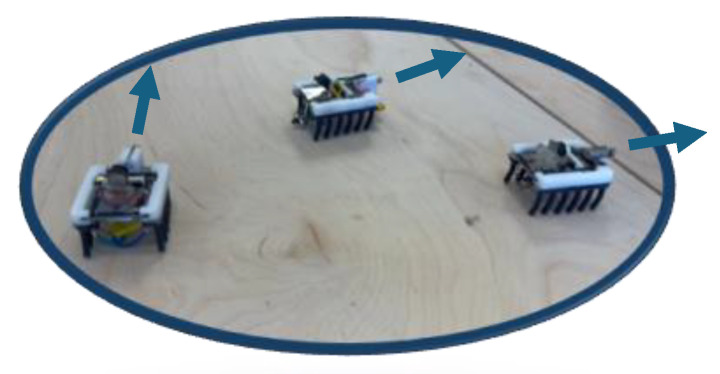
Swarm microrobot random-direction inspection of the infrastructure.

As for the steering of the microrobots, interfaces are programmed in the AR headset (HoloLens 2) and in the PC for steering the microrobots while providing visual feedback, for sending random direction commands at random times, and for displaying the images (Figure 6). The proposed system enables the swarm to perform random-direction inspection of the structures.

**Figure 7:**
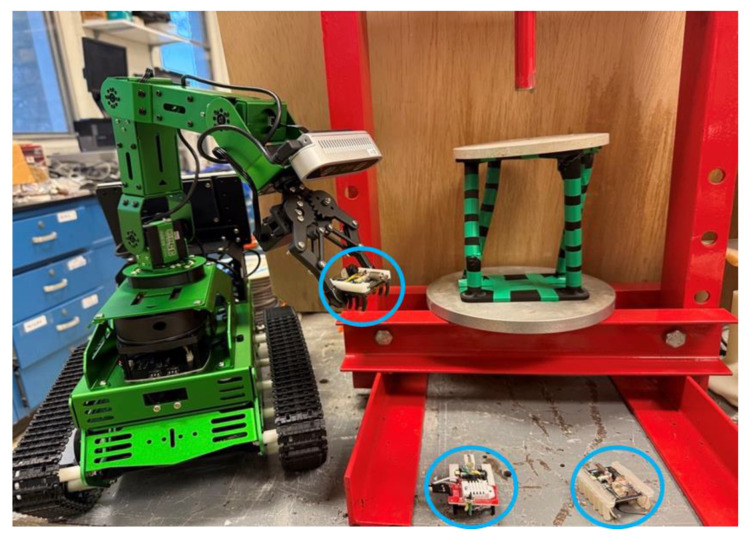
A robot with an arm placing the microrobots in the desired location.

The proposed microrobot swarm moves based on the stick–slip mechanism; therefore, its locomotion on different terrains is limited. For placing the robot in the location of interest, more robust robots with an arm are required to pick up and place the microrobots, as shown in Figure 7.

After placing the microrobots in the area of interest, the microrobots can steer to the desired confined space and adjust their angle of inspection toward the structure.

#### 2.1.2. Swarm Microrobot Semantic Crack Segmentation in Augmented Reality

While identifying cracks in confined spaces is challenging, early detection of cracks prevents disastrous collapses and costly repairs. Swarm microrobots can thoroughly search an area and broadcast images to an edge processor for the semantic segmentation of cracks and present them in AR to an inspector for analysis and assimilation.

To address real-world applications for the semantic segmentation of cracks through microrobotic inspections, a robust model based on the U-Net architecture [22] can capture varying crack sizes on a range of surfaces. Starting with a pre-trained model [23] using a diverse dataset from various sources [24,25,26,27,28,29,30,31,32], a more generalized robust model was developed for different materials and surfaces. Utilizing the Middle East Technical University dataset [33], the model continued to be fine-tuned using the database’s high-quality images in addition to augmentations for specific SHM applications. The perspective transformation data augmentation [34], in which the perspective is randomly warped using an acceptable intensity, helped to identify cracks regardless of the microrobot’s position, simulating the viewing of surfaces from a variety of distances and angles.

**Figure 8:**
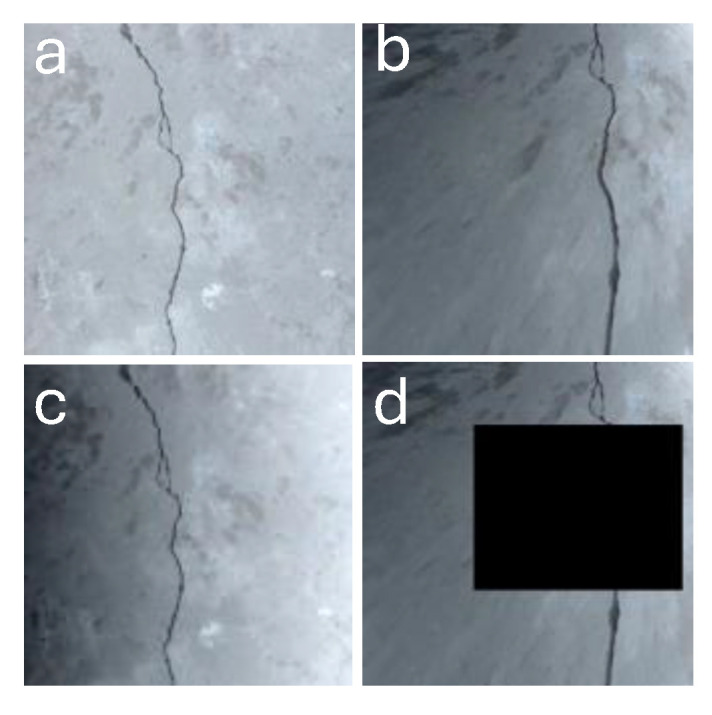
**Semantic crack segmentation techniques and challenges:** (**a**) Original image. (**b**) Perspective shift augmentation. (**c**) Gradient augmentation. (**d**) Occlusion.

Moreover, real-world applications were mimicked, using cases such as different lighting environments with shadows, random brightness gradients, and where a part of the surface might be covered (Figure 8). Based on this process, a new semantic segmentation model, with diverse datasets and image augmentations adapted to the specific requirements of the microrobot, is proposed.

For evaluating the effectiveness of the proposed CNN training methodology for microrobotic inspections, robustness to variations and poor-visibility scenarios were considered. The original dataset (non-augmented) of 458 image–mask pairs was used while reserving a test set in an 80–20 split. A second “augmented dataset” was constructed using the described methodology, reserving the same test split as in the original dataset.

Two models were trained with the identical architecture and training process, with one trained on the training split of the original dataset, and the second on the augmented dataset. Both were tested against both test splits to verify any gains in performance and robustness via the F-score, recall, and precision.

Table 1 shows the expected improvement in the test splits of the augmented training set without a drop in performance (or minor improvement) against the original dataset. This indicates that the proposed image augmentation process increases the robustness to varied scenarios that may be encountered in real-life applications.

#### 2.1.3. Swarm Microrobot Structural Deformation Analysis

**Figure 9:**
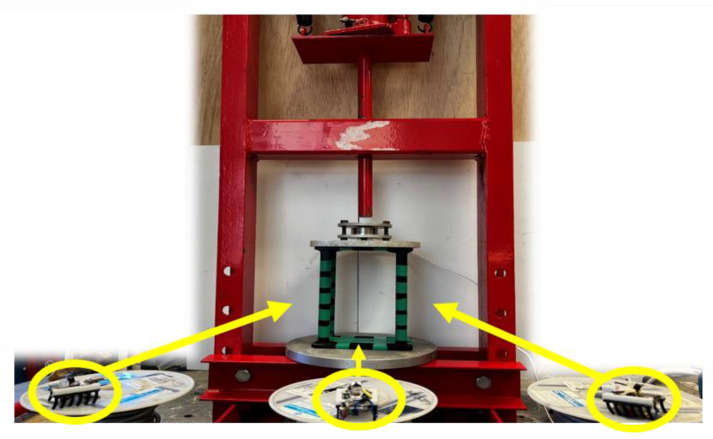
Swarm microrobot viewing the model from three different angles.

Monitoring changes in structural shapes can warn of and prevent catastrophic failures. Figure 9 shows how a swarm of three microrobots takes pictures of a 3D-printed model structure every second during a crush-to-failure experiment. The image data from the swarm is transmitted to an edge notebook computer that applies Canny edge detection [35] and calculates statistical parameters.

The parameters of Normalized Absolute Error (NAE) and the Peak Signal-to-Noise Ratio (PSNR) are calculated at every second to signal any pattern leading to failure. The parameters can be determined either between each consecutive image or between images from different angles.

#### 2.1.4. Culvert Multi-Robot Monitoring

Cyber–physical systems have a wide range of applications that require mobility, sensing, telemetry, cognition, human interface, and teaming. Considering the wide range of technologies involved, each aspect of the CPS would need special considerations based on the applications.

Culvert monitoring is challenging for humans due to the size and conditions of the culverts. Additionally, as the culverts are electromagnetically lossy, their inspection poses challenges for the wireless telemetry. In particular, long culverts and culverts with corners cause disruptions to the wireless signal. The signal attenuation and interference are not just limited to the Wi-Fi camera but also affect the mobile robot’s controller signal.

**Figure 10:**
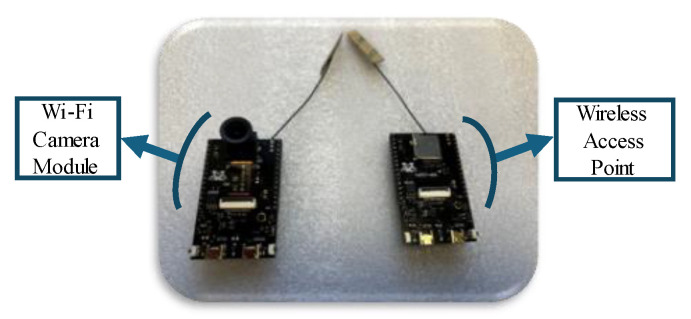
AMB82 mini-board and camera are used as a wireless access point and a Wi-Fi camera.

One approach is to leverage the waveguide behavior of a culvert and to utilize an antenna with a higher gain in the 5.8 GHz band, which has been observed to provide a greater operating range than the 2.4 GHz frequency [36]. Another possible solution for culvert telemetry is to place the dish reflector at the corners [10]. However, access to the corners may not always be possible. In this section, alternative approaches are proposed. For the experiment, culvert inspection vehicles such as HIVE 2.0 [36] can be modified using Wi-Fi-enabled cameras (AMB82-Mini, Realtek, Hsinchu, Taiwan)(as shown in Figure 10) to extend the range by their dual frequency or for their use in multi-robot configurations.

**Figure 11:**
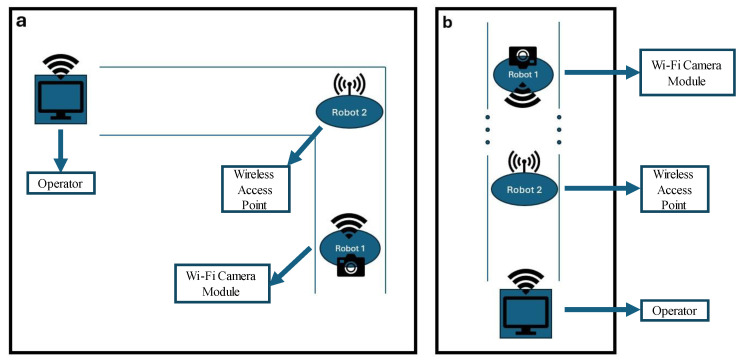
(**a**) Multi-robot monitoring of a culvert with a corner. (**b**) Long culvert inspection using the middle robot as the wireless access point.

The novel multi-robot approach demonstrated in Figure 11 includes using a middle robot as the wireless access point that stops at the corner or in the middle of a long culvert to assist in the transmission of the signal from the first robot to the operator conducting the inspection from outside the culvert.

#### 2.1.5. Wall and Pump Inspection Method

Monitoring the deformation of structures over time can assist in preventing their failure. Robotic systems integrated with LiDAR or depth cameras can be utilized to perform this task. Similarly, acoustic data can be captured in hard-to-reach areas for detecting early signs of defects.

As the QRD is a versatile robotic system that can be mounted with sensors, microphones, and cameras, it can be used for the inspection of devices and structures.

**Figure 12:**
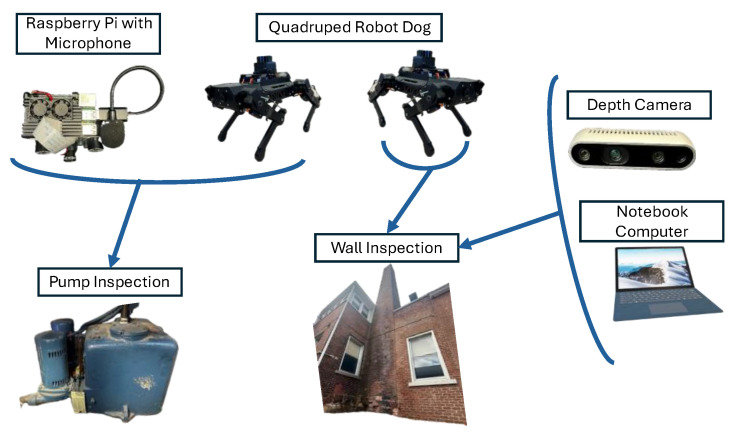
Schematic of the hardware utilized for pump and wall inspections.

Figure 12 shows a schematic of the hardware used to conduct inspections of walls and devices commonly used in buildings.

**Figure 13:**
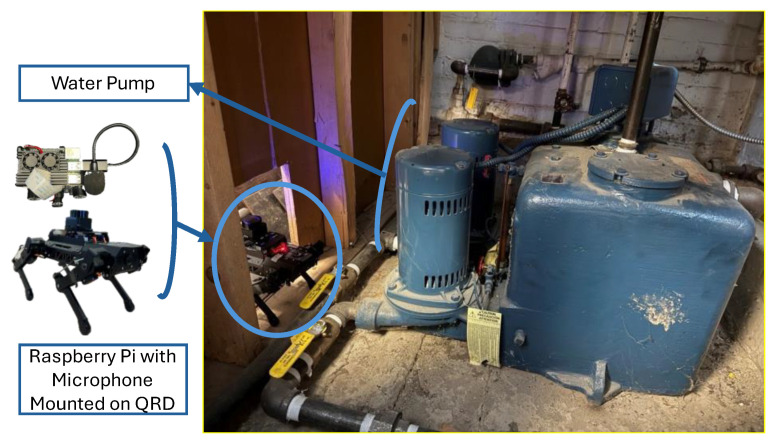
Quadruped robot dog (Puppy Pi Pro Hiwonder) configuration for inspecting the water pump.

Fath et al. [37] used a quadruped robot dog (Puppy Pi Pro Hiwonder) to carry out a visual inspection using AR and to compare the acoustic data of a pump. The pump is in a small room with a small rectangular opening for access to low-light conditions. A microphone (Figure 13) was used to analyze the pump acoustic data over a year.

Similarly, a new test was conducted to compare a damaged and an undamaged pump using acoustic data. In this experiment, the case of the damaged pump was considered by attaching a rattling mass and spring to the body of the pump. The undamaged pump was considered without adding any mass and spring to the system. The acoustic data in both cases were analyzed when the pump was turned on and off and the processed data was compared through the spectrograms plotted in MATLAB R2023b.

**Figure 14:**
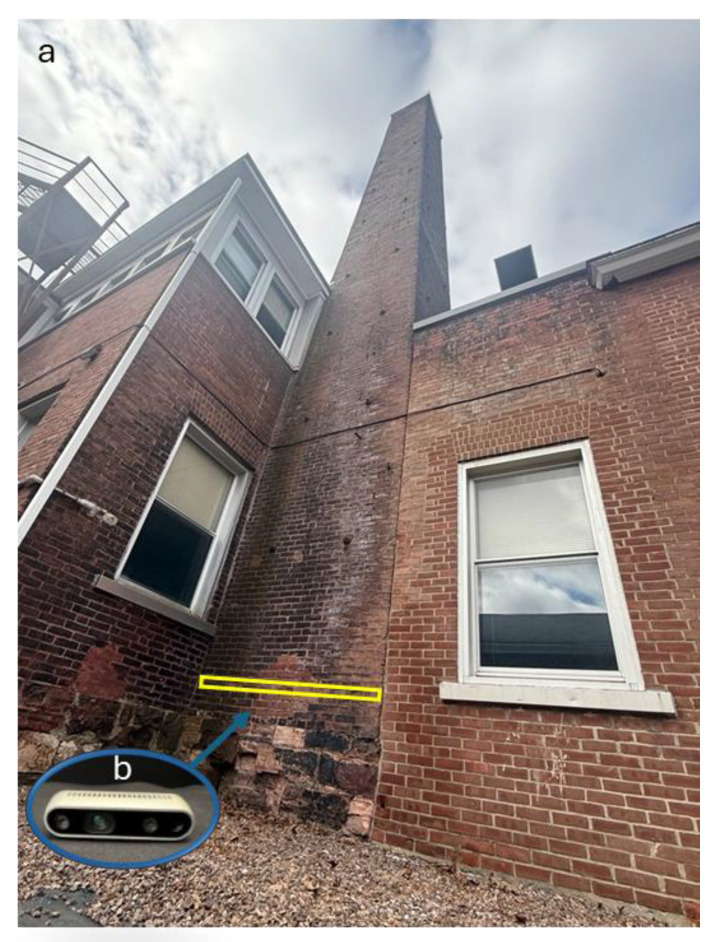
(**a**) Old chimney wall outside Perkins Hall at the University of Vermont. (**b**) Intel RealSense D435 depth camera utilized for the wall inspection.

In addition to the inspection of the walls with a LiDAR unit mounted on the QRD [37], the same wall of an old chimney outside the Perkins Hall at the University of Vermont was inspected again using the Intel RealSense D435 depth camera (Intel, Santa Clara, CA, USA) (Figure 14b) connected to a notebook computer with a GPU using RTAB-Map v0.20.16 [38]. The radius of curvature was calculated using the curveFitter toolbox in MATLAB R2023b that fitted a second-degree polynomial to the data captured by the depth camera.

## 3. Results

In this section, some of the applications of the proposed CPS framework are experimented with using function tests. The results are elaborated based on the requirements of the applications.

### 3.1. Swarm Microrobot Semantic Segmentation of Cracks in Augmented Reality

**Figure 15:**
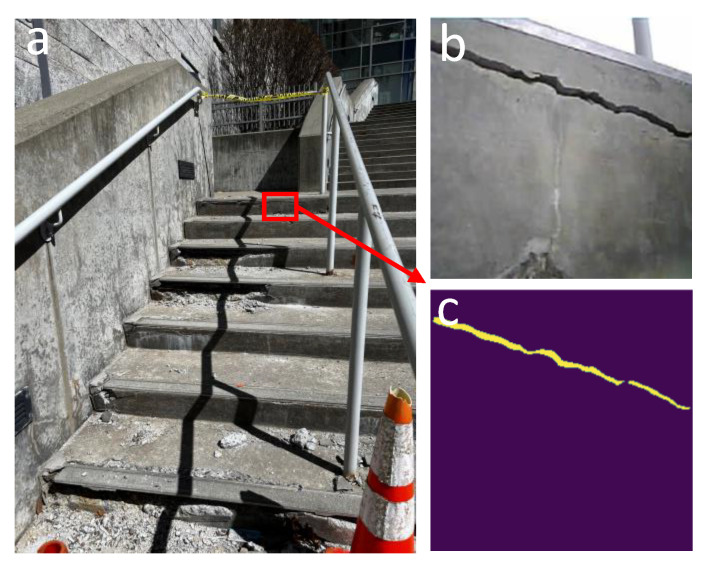
(**a**) Cracks located on the reinforcing steel. (**b**) Image of a crack captured by the left microrobot. (**c**) Semantic segmentation of the crack.

To verify the effectiveness of the proposed swarm semantic segmentation of cracks in AR, a test was conducted on the cracks of concrete stairs with damage that exposed the reinforcing steel (Figure 15).

**Figure 16:**
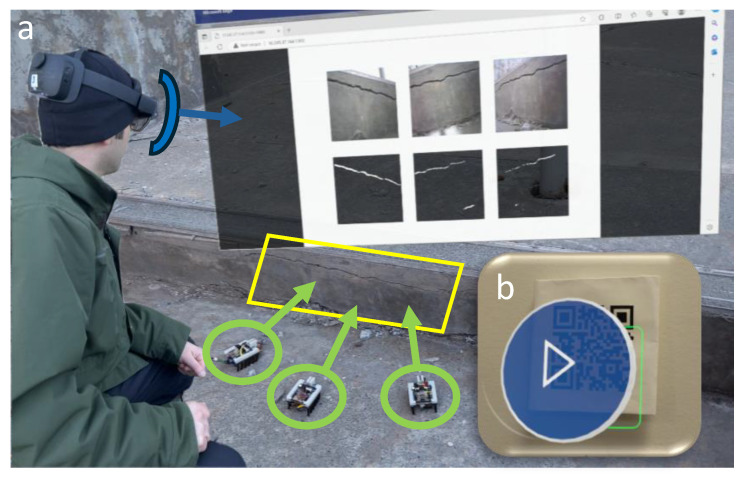
(**a**) AR semantic segmentation of cracks using the swarm of microrobots. (**b**) QR code for accessing the Flask server.

The experiment uses the images captured from different angles by the microrobots, applies the model, and uploads the results to the Flask server. For viewing the generated masks with the original images, an AR headset is used which projects a digital overlay of the images by scanning a QR code linked to the Flask server (Figure 16).

**Figure 17:**
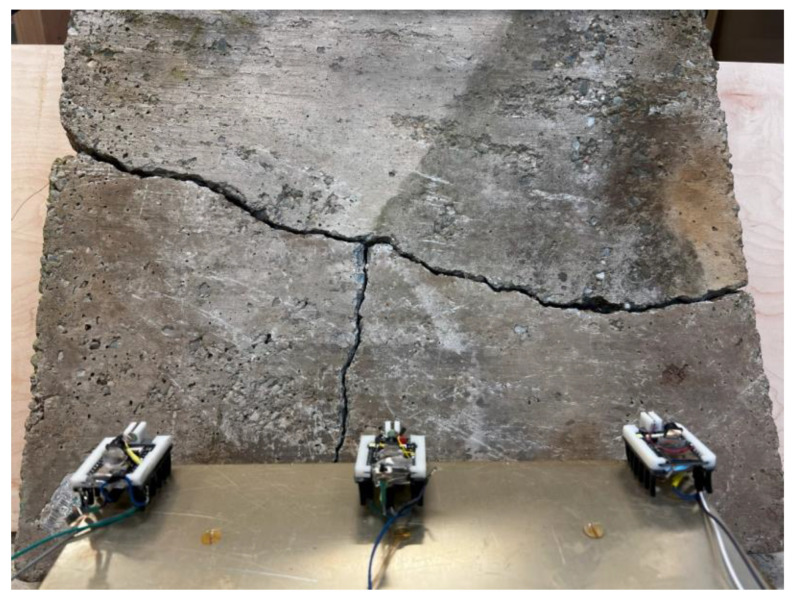
Microrobots capture front-viewed images of a long crack.

The same process was repeated in the lab with a concrete slab having a long “T”-shaped crack (Figure 17).

**Figure 18:**
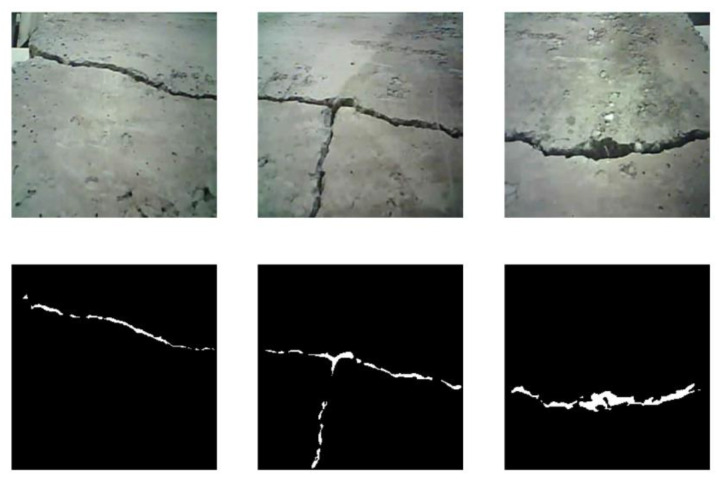
The images of the cracks with their corresponding masks.

In this experiment, the goal is to take pictures of sections of the crack from different microrobots in a swarm and to merge them for semantic segmentation of the long crack in AR. Figure 18 shows each image taken by the microrobots and their semantic segmentation mask.

**Figure 19:**
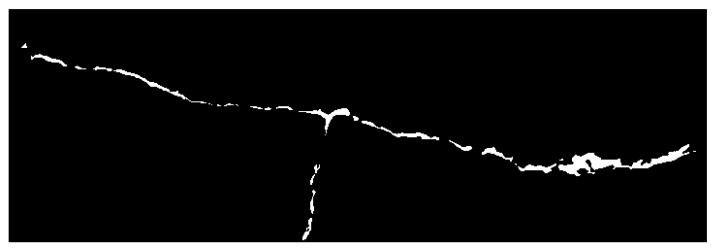
The masks merged together to represent the long crack.

The corresponding masks were merged side by side, generating the long crack mask depicted in Figure 19.

**Figure 20:**
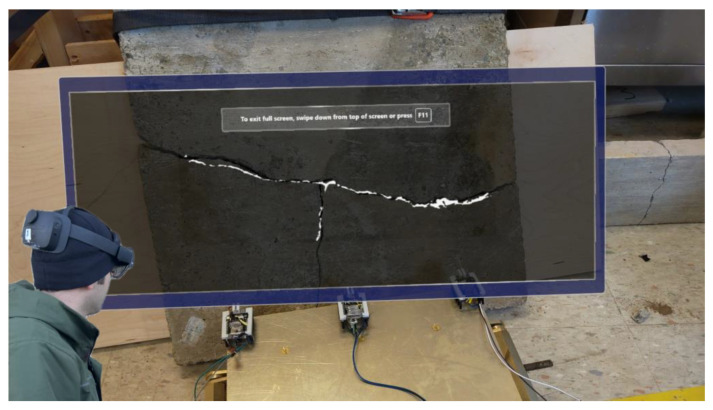
AR semantic segmentation of a long crack using the swarm of microrobots.

As the masks have a small file size, they can be accessed efficiently in a cyber–physical system framework by scanning a QR code, containing the IP address of the Flask server, viewed by the operator wearing an AR headset (Figure 20).

### 3.2. Swarm Microrobot Structural Deformation Analysis via Computer Vision

For enhanced deformation detection, the swarm of three microrobots started capturing images from three angles during the crush-to-failure test. During the experiment, an edge notebook computer was running a Python 3 code on JupyterLab v3.2.1 using Anaconda Navigator. The program captured images from all three microrobots, applied the edge detection algorithm, and calculated the statistical parameters.

**Figure 21:**
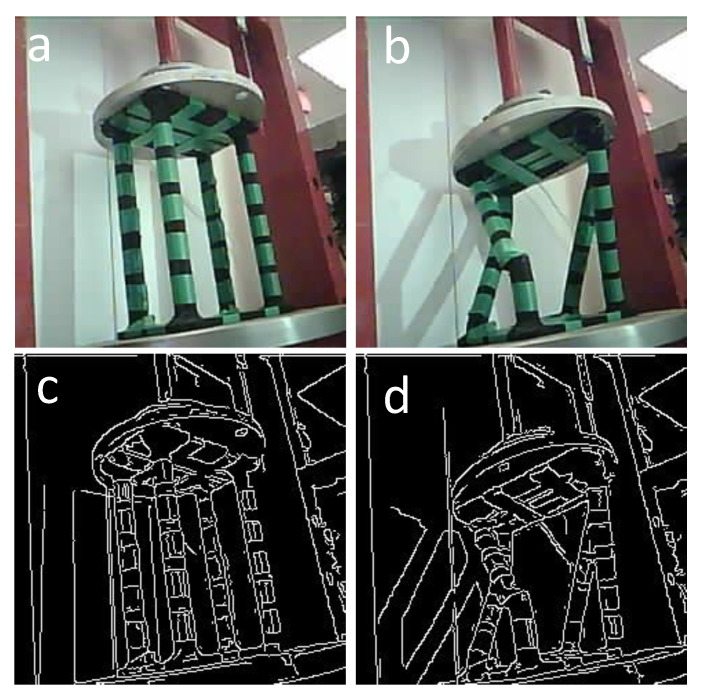
(**a**) Original image of the initial stage captured by the left microrobot. (**b**) Original image of the final stage captured by the left microrobot. (**c**) Edge-detected image of the initial stage captured by the left microrobot. (**d**) Edge-detected image of the final stage captured by the left microrobot.

The goal of using a swarm for this experiment is to see if different views of a collapsing structure can provide any predictive valuable information about the structure failure. Additionally, conducting the task in a swarm makes the system more robust. As an example, if a microrobot fails to perform the function, the goal of the inspection is still satisfied, which, in this case, occurred as the middle microrobot’s battery died during the experiment. However, the left and right microrobots continued capturing the whole process. Figure 21 shows the left microrobot images of the initial and final state during the crush-to-failure test with their corresponding edge-detected images.

**Figure 22:**
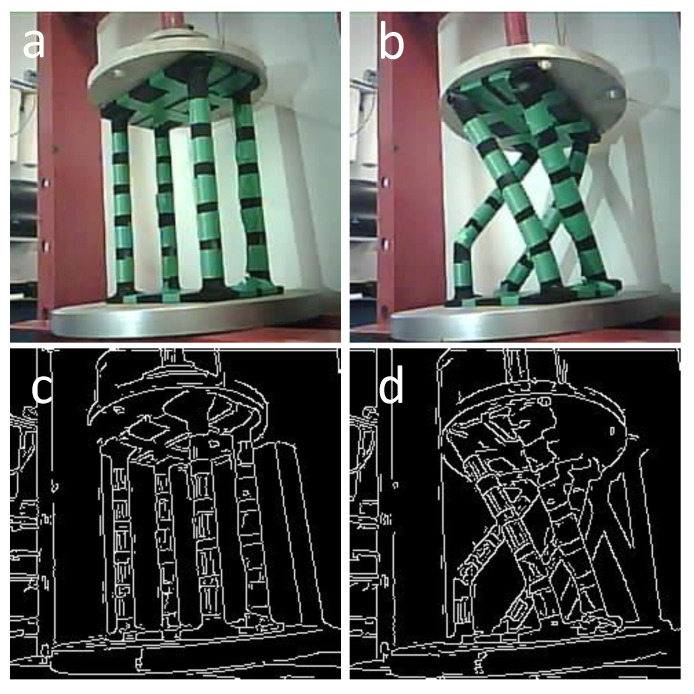
(**a**) Original image of the initial stage captured by the right microrobot. (**b**) Original image of the final stage captured by the right microrobot. (**c**) Edge-detected image of the initial stage captured by the right microrobot. (**d**) Edge-detected image of the final stage captured by the right microrobot.

Similarly, the right-side microrobot captured the same images from the right view, as depicted in Figure 22.

**Figure 23:**
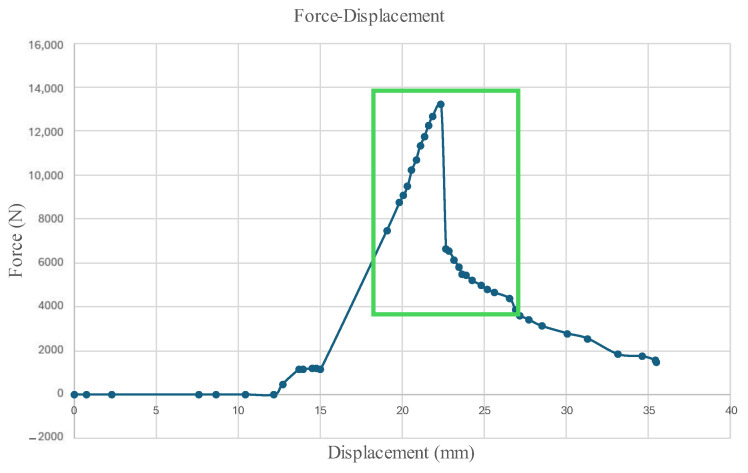
Force–displacement graph of the crush-to-failure test.

The force–displacement graph is plotted in Figure 23, where the information is recorded manually by the operator reading the information. The force captured by the load cell and the displacement were shown on the caliper attached to the hydraulic press and the moving platform.

**Figure 24:**
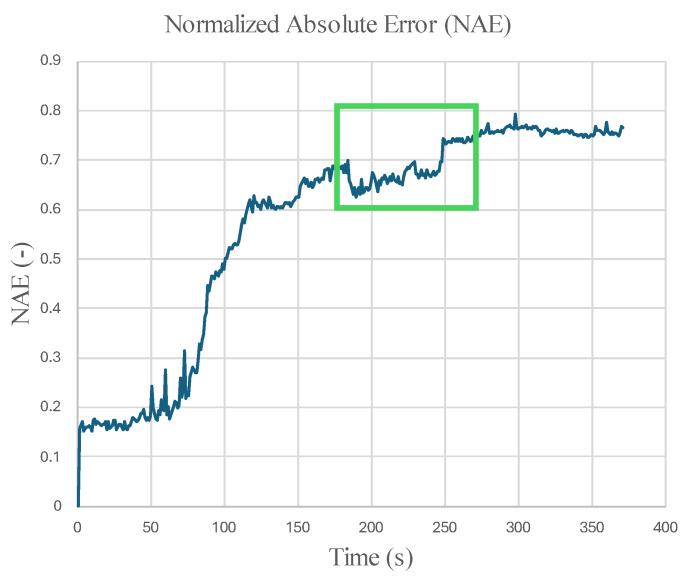
NAE between the first image taken by the left microrobot and each consecutive image. Green box indicates time with significant change.

**Figure 25:**
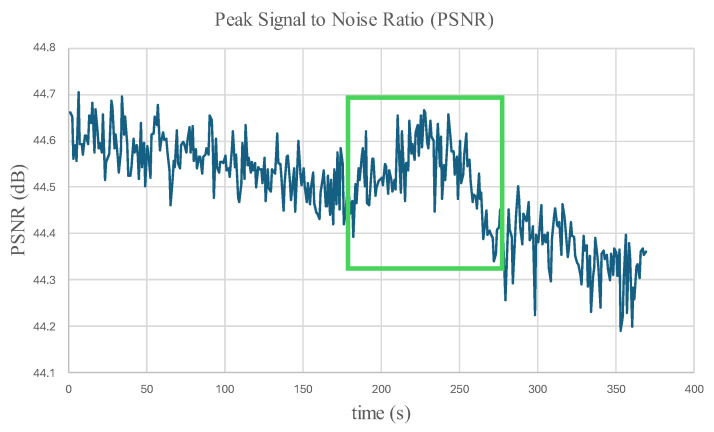
PSNR between images taken by the right and left microrobots. Green box indicates time with significant change.

Figure 24 and Figure 25 display the Normalized Absolute Error (NAE) (Equation (1) [39]) between the first image and each consecutive new image, and the Peak Signal-to-Noise Ratio (PSNR) (Equation (2) [40]) between corresponding images captured by the left and right microrobots with respect to time. The green box encloses changes that indicate precursors to failure. The parameters are calculated based on the grayscale pixel intensity of the edge-detected images.

(1) $$NAE=\frac{\sum_{1}^{m}\sum_{1}^{n}\left|I_{1}-I_{j}\right|}{\sum_{1}^{m}\sum_{1}^{n}\left|I_{1}\right|},\;\;j=2,3,4,\ldots ,t_{f}$$

where $I_{j}$ is the pixel intensity of the jth consecutive image, $t_{f}$ is the final time, n is the number of rows, and m is the number of columns of pixels in the image.

(2) $$PSNR=20{log}_{10}\frac{{MAX}_{intensity}}{\sqrt{MSE}},\;\;MSE=\frac{\sum_{1}^{m}\sum_{1}^{n}{\left|I_{L}-I_{R}\right|}^{2}}{n\times m}$$

where ${MAX}_{intensity}$ is the maximum pixel intensity, and MSE is the mean squared error between the pixel intensities of the left ($I_{L}$) and right ($I_{R}$) microrobots.

In addition to the robustness of inspection with microrobot swarms, the changes in Figure 25 reveal the benefits of using multi-view swarm inspection to enhance structural failure detection.

**Figure 26:**
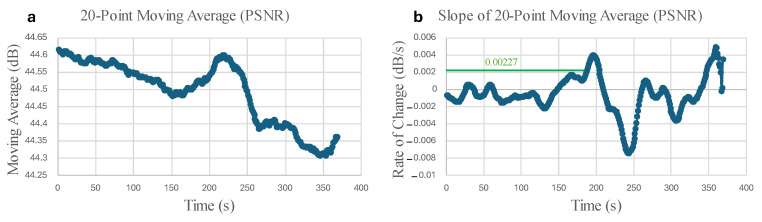
(**a**) The 20-point moving average of the PSNR in terms of time. (**b**) The 20-point slope of the 20-point moving average, showing the rate of change in terms of time.

From Figure 26, it can be interpreted that if the 20-point rate of change in the 20-point PSNR moving average changes more than the threshold of 0.00227 dB/s in this experiment, this indicates the possibility of failure.

### 3.3. Wall and Pump Inspection Results

**Figure 27:**
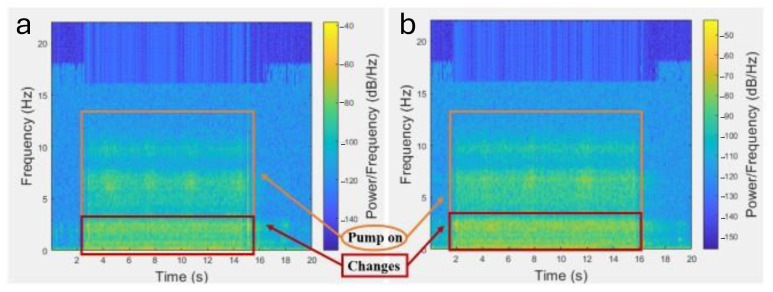
(**a**) Acoustic analysis of the pump without and (**b**) with an added mass and spring.

In the case of the undamaged pump and of the simulated damaged pump (which was created by adding a rattling mass and spring to the pump), the results can be compared using the spectrogram of the acoustic data from when the water pump was turned on and off. Figure 27a,b demonstrate power changes at low frequencies in the spectrogram between the given cases, which, in real-world scenarios, could indicate damage to the components of the pump.

**Figure 28:**
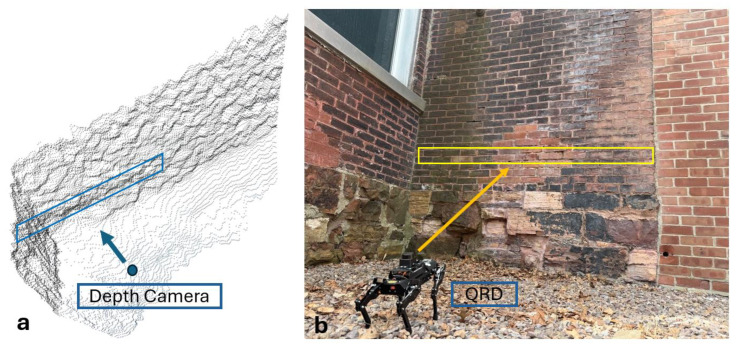
(**a**) Rendering of the point cloud of a wall near the chimney tower outside Perkins Hall, University of Vermont, created by the RTAB software using the RealSense depth camera. (**b**) The QRD inspecting the wall using LiDAR.

For the wall inspection, the curve fitting was performed by MATLAB R2023b on the point clouds of the depth camera, and the radius of curvature was determined to have a value of 15.48 m, which was previously 11.869 m in 2014 [41]. Due to masonry repairs, the wall appeared less curved than in 2014. The depth camera results are unlike the results with the quadruped robot dog (QRD) using LiDAR, with a value of 20.22 m, and the manual measurements of the wall curvature, with a value of 20.6672 m [37]. A comparison of the results shows that using the LiDAR on the QRD provides more accurate results with reference to the manual measurements. Figure 28 shows the point clouds captured by the RealSense depth camera (Intel, Santa Clara, CA, USA) in the RTAB-Map v0.20.16 software [38].

### 3.4. Multi-Robot Culvert Monitoring

**Figure 29:**
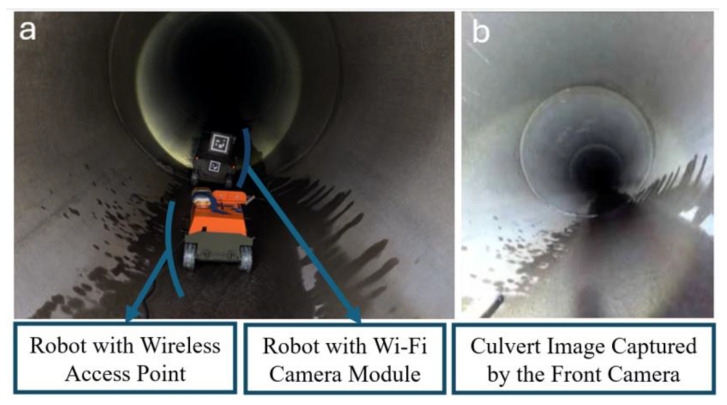
(**a**) Culvert monitoring using a multi-robot system. (**b**) Image captured by the front robot’s camera transmitted back to the operator.

Functioning of the proposed multi-robot solution for monitoring long culverts was tested in a culvert in Vermont, USA, as demonstrated in Figure 29.

In this test, the front robot carries the Wi-Fi-enabled camera and transmits the video back to a similar board programmed as an access point on the rear robot. Hence, the video feed is accessible for inspection of the culvert from outside the culvert by the operator connected to the access point on the rear robot.

**Figure 30:**
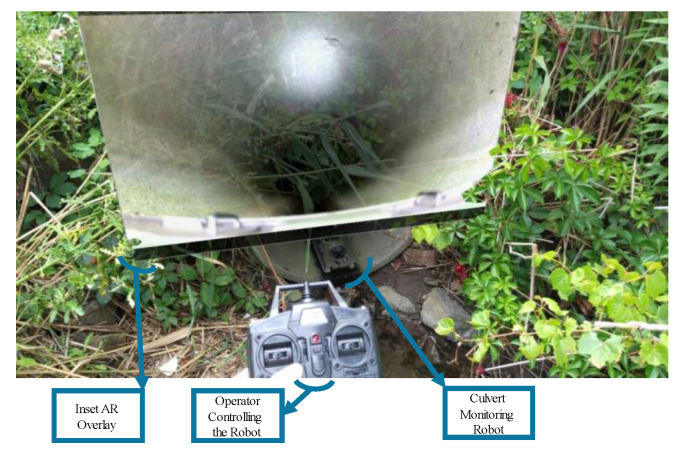
Digitally rendered visual data projected onto the operator’s AR headset while controlling the robot for culvert inspection.

The operator can access the data using devices such as a notebook computer, a phone, and an augmented reality headset. Figure 30 demonstrates this capability by showing a digitally rendered overlay of the visual data captured by the robot inspecting the culverts.

## 4. Discussion

Despite growing advancements in the technology of robotics, sensing, and inspection, the lack of cyber–physical approaches for implementing the CPS in challenging SHM applications has led to issues in detecting and preventing the catastrophic failure of structures.

Fabricating a swarm of microrobots with random-direction inspections allows for the comprehensive monitoring of confined spaces, and edge processing of the received images could enhance the damage detection and lead to automation of the process.

The previous models for the semantic segmentations of cracks are not applicable to the specific view angle of the microrobots and the lighting conditions of the confined spaces. Thus, image augmentations are necessary for adapting the models to the specific requirements of the microrobots’ inspection.

The swarm microrobots’ function tests for the semantic segmentations of cracks were successful by capturing images from different angles, performing the semantic segmentation, and uploading the results to a Flask server for the AR operator’s inspection. Additionally, edge processing for the swarm of microrobots is capable of merging the photos together to create a mask of the long crack. This generates a comprehensive view of the crack from several images and provides small-sized data for transfer to the AR user. This process provides an efficient data transmission method in the case of a weak signal.

As monitoring for the structural deformations and calculation of the statistical parameters are based on edge detection, tapes with different colors are added to the 3D-printed part to enhance the performance of the algorithm. The significant changes before the failure of the 3D-printed part in the PSNR graph demonstrate the advantage of using swarm microrobots for SHM. The experiment verified the fact that the swarm SHM increases the reliability of the CPS. As an example, in the given experiment in this study, the middle microrobot battery was depleted sooner than the others, but the results of the remaining two microrobots still demonstrated significant changes in the PSNR graph before the failure of the part.

Although studying deformations in the laboratory model does not fully represent the live loads [42,43,44] present in in-service structures, the current process demonstrates the successful implementation of swarm monitoring of deformations in structures. Examples of cases of increasing load on structures are snow accumulation and foundational damage, where part of the structure undergoes excessive loading. For structures experiencing varying loads, a similar CPS can be applied while automating the process by using appropriate fixed time intervals based on the type of structure. Nonetheless, further statistical analysis should be conducted on the deformation of each specific structure to determine the threshold values indicating the severe underlying problem.

The device inspection performed on a water pump shows the functioning of the acoustic monitoring of such devices by using robots in confined spaces. The process can be automated to inspect the same device at fixed time intervals to compare the changes and to prevent device failure. A similar approach is useful in monitoring the walls of structures by using robots equipped with LiDAR and depth cameras to analyze the trend of deformation and to avoid catastrophic failures.

Furthermore, for applications with network connectivity issues such as culverts, the proposed modular swarm robot inspection method proposes a robust low-cost inspection solution that assists in the maintenance of the culverts without the risk of any human entering the unstable or hard-to-reach culverts to conduct the inspection.

Future works include metrics and evaluation methods for this framework to provide a basis for analyzing the performance of the CPS frameworks.

## 5. Conclusions

In this study, several aspects of cyber–physical systems for challenging SHM applications were investigated. The HeSARIC framework, which includes a swarm of robots, sensors, microrobots, and AR, was proposed for solving various issues in confined-space monitoring and to enhance damage detection, data efficiency, network connectivity, human interaction, and robustness. Functioning of the CPS was demonstrated through several application tests, and their specific requirements for the applications were elaborated.

Possible applications of this framework are the detection of deformations of structures in confined spaces, swarm semantic segmentation of cracks in AR, wall deformation analysis, pump acoustic inspection, and culvert monitoring while providing an AR interface for swarm robots and sensors.

## Figures and Tables

**Table 1:** Comparison of the proposed semantic segmentation model with the original model.

Model	Test Set	F-Score	Precision	Recall
Original training set	Original	0.156	0.120	0.312
	Augmented	0.057	0.058	0.044
Augmented training set	Original	0.164	0.116	0.400
	Augmented	0.103	0.048	0.216

## Data Availability

The original contributions presented in the study are included in the article; further inquiries can be directed to the corresponding author.
